# Inferring demographic and selective histories from population genomic data using a 2-step approach in species with coding-sparse genomes: an application to human data

**DOI:** 10.1093/g3journal/jkaf019

**Published:** 2025-01-30

**Authors:** Vivak Soni, Jeffrey D Jensen

**Affiliations:** School of Life Sciences, Center for Evolution & Medicine, Arizona State University, Tempe, AZ 85281, USA; School of Life Sciences, Center for Evolution & Medicine, Arizona State University, Tempe, AZ 85281, USA

**Keywords:** demography, distribution of fitness effects, background selection, selective sweeps, genome scans, genetic hitchhiking

## Abstract

The demographic history of a population, and the distribution of fitness effects (DFE) of newly arising mutations in functional genomic regions, are fundamental factors dictating both genetic variation and evolutionary trajectories. Although both demographic and DFE inference has been performed extensively in humans, these approaches have generally either been limited to simple demographic models involving a single population, or, where a complex population history has been inferred, without accounting for the potentially confounding effects of selection at linked sites. Taking advantage of the coding-sparse nature of the genome, we propose a 2-step approach in which coalescent simulations are first used to infer a complex multi-population demographic model, utilizing large non-functional regions that are likely free from the effects of background selection. We then use forward-in-time simulations to perform DFE inference in functional regions, conditional on the complex demography inferred and utilizing expected background selection effects in the estimation procedure. Throughout, recombination and mutation rate maps were used to account for the underlying empirical rate heterogeneity across the human genome. Importantly, within this framework it is possible to utilize and fit multiple aspects of the data, and this inference scheme represents a generalized approach for such large-scale inference in species with coding-sparse genomes.

## Introduction

Genetic variation is a fundamental concern of population genetics. Prior to the advent of next-generation sequencing, a dominant debate within the field was centered on whether levels of genetic variation were expected to be minimal or substantial (known as the *classical*/*balanced* debate; see Crow 1987; Lewontin 1987). Selection was assumed as the dominant process in both cases, be it purifying selection depressing levels of variation or balancing selection maintaining polymorphism (Dobzhansky 1955). Despite molecular evidence confirming plentiful levels of genetic variation, Motoo Kimura's *The Neutral Theory of Molecular Evolution* (Kimura 1968, 1983) instead posited that observed variation was largely a consequence of genetic drift, that is, of neutral alleles segregating in the process of drifting toward fixation or loss. This hypothesis—that neutral rather than selective processes can explain the majority of observed variation—has since been largely corroborated (as reviewed in Jensen *et al*. 2019).

However, quantifying the precise roles of selective and neutral processes in shaping observed levels of variation—and disentangling their individual effects—remains an ongoing challenge due to the similar manners in which multiple evolutionary processes affect patterns of variation. One notable example is the extent to which neutral population growth, background selection (BGS; Charlesworth *et al*. 1993), and recurrent selective sweeps (Maynard Smith and Haigh 1974) can all skew the site frequency spectrum (SFS, the distribution of allele frequencies) toward rare alleles (Kim 2006; Jensen *et al*. 2007; Nicolaisen and Desai 2012, 2013; Ewing and Jensen 2016; Johri *et al*. 2021; Soni *et al*. 2023; and see review of Charlesworth and Jensen 2021, 2024). The effects of these processes are further modified by genomic heterogeneity in mutation and recombination rates in often complex ways (Soni, Pfeifer, *et al.* 2024). Therefore, if one wishes to quantify the strength and frequency of rare and episodic processes such as positive selection, one must first construct an evolutionarily appropriate baseline model that accounts for the effects of constantly occurring processes including genetic drift as modulated by historical population size changes, as well as the effects of purifying selection and BGS resulting from the removal of deleterious mutations (Bank *et al*. 2014; Johri, Aquadro, *et al.* 2022), all while accounting for underlying mutation and recombination rate variation. Failure to account for these processes is likely to lead to misinference, particularly in light of the fact that many commonly studied populations and species are thought to have experienced not only population growth but also recent and severe population bottlenecks [e.g. humans (Gutenkunst *et al.* 2009; Gravel *et al.* 2011; Excoffier *et al.* 2013), nonhuman primates (Soni, Terbot, Versoza, *et al.* 2024; Terbot *et al*. 2025), and *Drosophila melanogaster* (Li and Stephan 2006), as well as a variety of human pathogens (Irwin *et al.* 2016; Sackman *et al*. 2019; Jensen 2021; Morales-Arce *et al.* 2022)], a demographic history that is itself often strongly confounded with selective sweeps (Barton 1998; Poh *et al*. 2014; Matuszewski *et al*. 2018; Harris and Jensen 2020; Charlesworth and Jensen 2022; Jensen 2023).

Constructing an evolutionarily appropriate baseline model for a given population will therefore require inferring both a demographic history and the distribution of fitness effects (DFE) of new mutations. However, because population history can confound DFE inference, it is necessary to correct for the demographic history of the population in question (Eyre-Walker and Keightley 2007; Boyko *et al*. 2008). The most commonly used class of approaches are based on a framework in which demographic inference is performed on putatively neutral sites, before utilizing that demographic history for DFE inference on functional sites (Eyre-Walker and Keightley 2007; Boyko *et al*. 2008; Galtier 2016; Tataru and Bataillon 2020; and see review of Johri, Eyre-Walker, *et al*. 2022). Eyre-Walker and Keightley (2007) obtained the first computationally inferred DFE estimates using this approach, and further work incorporated a beneficial class of mutations into the inferred DFE (Boyko *et al*. 2008; Eyre-Walker and Keightley 2009; Schneider *et al*. 2011; Galtier 2016).

Notably, this type of 2-step approach is often performed on functional regions under the assumption that all sites are independent and unlinked, and that synonymous sites are selectively neutral. However, these synonymous sites are likely experiencing BGS effects (Charlesworth *et al*. 1993) due to linkage with directly selected and adjacent nonsynonymous sites, resulting in a skew in the SFS and thus misinference; in particular, these BGS effects are often misinterpreted as population growth (Ewing and Jensen 2014; Johri *et al*. 2021; and see review of Johri, Eyre-Walker, *et al*. 2022). More generally speaking, there is indeed substantial evidence that the effects of selection at linked sites may be widespread across the genomes of many commonly studies species (see reviews of Cutter and Payseur 2013; Charlesworth and Jensen 2021). Although recent work has shown that DFE inference is relatively robust to the biasing effects of selection at linked sites (Kim *et al*. 2017; Huang *et al*. 2021), that is not the case for demographic inference (Messer and Petrov 2013; Nicolaisen and Desai 2013; Ewing and Jensen 2016; Schrider *et al*. 2016; Johri *et al*. 2021). It is also noteworthy that these 2-step approaches are generally constrained to relatively simple population histories utilizing a 2-epoch model (Williamson *et al*. 2005; Keightley and Eyre-Walker 2007; Kousathanas and Keightley 2013).

The second class of methods involves using forward-in-time simulations (e.g. in SLiM; Haller and Messer 2023) to jointly and simultaneously infer population history with the DFE in an approximate Bayesian (ABC) framework (see Beaumont *et al*. 2002), as proposed by Johri *et al*. (2020). Within this simultaneous inference scheme, it is neither necessary to assume a priori the neutrality of synonymous sites nor is it necessary to assume independence among sites; as such, BGS can be directly modeled and incorporated. While 2-step methods commonly infer a continuous distribution for the DFE, this ABC framework infers a number of discrete DFE categories for various ranges of 2*N*_e_*s*, the population-scaled selection coefficient, where *N*_e_ is the effective population size and *s* is the strength of selection acting on new mutations within the DFE category of interest. The main drawback of such methods is that they are computationally expensive given the large parameter space that must be explored when jointly inferring both demographic and DFE parameters. As such, the inferred demographic models have thus far been limited to single-step size changes in which the ancestral and current population sizes, as well as the timing of size change, are inferred (Johri *et al*. 2020, 2023). Importantly however, in coding-dense and/or nonrecombining species in which sufficiently neutral, unlinked genomic regions may not exist in the genome (thus precluding the needed neutral demographic inference underlying 2-step approaches), this simultaneous inference framework remains the only viable approach (e.g. Howell *et al*. 2023; Terbot, Cooper, *et al*. 2023; Terbot, Johri, *et al*. 2023; Soni, Terbot, *et al*. 2024).

It thus stands as an outstanding evolutionary inference question of how best to accurately infer a necessarily complex and realistic demographic model, along with a realistic DFE governing functional genomic regions, all while accounting for the variety of discussed potential biases. Here we have investigated a modified 2-step approach applied to human populations, in which the population history was inferred using nonfunctional regions sufficiently distant from functional sites in order to avoid BGS effects, DFE inference was then performed on exonic regions accounting for BGS effects and conditional on the demographic history inferred in step 1, and mutation and recombination rate maps were utilized to account for the modulating effects of this underlying heterogeneity. By inferring these parameters separately, a more biologically realistic population history was possible accounting for the complexities of population size change, structure, and migration patterns in these studied human populations, while the utilization of these distant nonfunctional regions allowed for the reduction or elimination of the biasing effects of BGS on demographic inference. While a number of coalescent and diffusion approximation-based approaches would be easily incorporated into our framework (e.g. Gutenkunst *et al*. 2009; Excoffier *et al*. 2013; Jouganous *et al*. 2017; Wang *et al*. 2020), this approach—like the ABC approach of Johri *et al*. (2020) and Johri, Aquadro, *et al*. (2022)—has the benefit of utilizing various aspects of population genomic data, including the SFS, associations between variants (linkage disequilibrium, LD), and population differentiation.

As human populations have naturally been highly studied, with numerous published demographic models, we here provide an optimized and well-fitting 4-population demographic model for the Out-of-Africa (OOA) expansion. Conditional on this model, we additionally optimized a DFE using genic regions, fitting both levels and patterns of polymorphism and divergence, and finding consistency with the recent DFE estimates of Johri *et al*. (2023), with optimization performed via grid search. Finally, we have evaluated the degree to which positively selected mutations may be identifiable within the context of this fit model. This work thus provides a valuable and improved framework for evolutionary inference in coding-sparse genomes and for the construction of evolutionary baseline models in such species.

## Materials and methods

### Data

This study was based on the GRCh37 human reference genome, with SNP data and accessibility masks obtained from 1000 genomes variant call format and bed files, respectively (The 1000 Genomes Project Consortium 2015). The data were split into continental populations, informed by levels of admixture, as determined by The 1000 Genomes Project Consortium (2015). The total number of samples from each of the 4 considered populations was: African—99; European—502; East Asian—104; and South Asian—489. We obtained recombination and mutation rate maps from Halldorsson *et al*. (2019) and Francioli *et al*. (2015), respectively, gene annotations from NCBI (Sayers *et al*. 2022), and ancestral sequences from the 6-way EPO alignments available from Ensembl (Flicek *et al*. 2014; Cunningham *et al*. 2022), and we identified conserved elements via the 100-way PhastCons score (Siepel *et al*. 2005; Pollard *et al*. 2010). See Supplementary Table 1 for links to all downloaded data.

### Selecting nonfunctional regions for demographic inference

For demographic inference, we identified nonfunctional regions of the human genome that were at a distance of at least 10 kb from the nearest functional region [as per the NCBI GFF file (Sayers *et al*. 2022)]. We then masked these regions using both strict accessibility masking (The 1000 Genomes Project Consortium 2015) and conserved element masks [i.e. with a phastCons score > 0 (Siepel *et al*. 2005; Pollard *et al*. 2010), in order to remove sites potentially experiencing purifying selection and generating BGS effects, e.g. binding sites (Simkin *et al*. 2014)]. Across each region, we calculated mean recombination and mutation rates, with any regions lacking this information being removed. Finally, we set a minimum length threshold of 15 kb to ensure that regions were long enough to reliably calculate summary statistics. Following these steps, we were left with a total of 146 nonfunctional regions. Finally, we used the *B* maps of McVicker *et al*. (2009) to compare the distribution of *B* values (i.e. the estimated reduction in diversity attributed to BGS by McVicker *et al*. 2009) to the distribution of our nonfunctional regions. For this analysis, we lifted over *B* map coordinates from the hg18 human genome assembly to the GRCh37 assembly using the UCSC liftover tool (Karolchik *et al*. 2003). Supplementary Fig. 1 provides plots of this comparison, as well as the distributions of region lengths, SNPs, and mutation and recombination rates for our set of curated nonfunctional regions.

### Selecting exons for DFE inference

We used the set of exons curated by Johri *et al*. (2023), although our focus was on the exonic regions only, as opposed to the exons and the neighboring intergenic regions. Because we used different recombination and mutation rate maps (as described in the “*Data*” section above), we recalculated mean rates across the 465 exonic regions, removing regions for which we did not have rate information, leaving a total of 397 exonic regions.

### Calculating empirical summary statistics

We calculated summary statistics for each population sampled using the python library for libsequence, Pylibseq v0.2.3 (Thornton 2003), except for *F*_ST_ which was estimated using scikit-allel (Miles *et al*. 2024). The number of segregating sites and *F*_ST_ were calculated per site, while Tajima's *D* (Tajima 1989) and mean *r*^2^ were calculated over 10 kb windows for each nonfunctional region and per region for each exon.

Exonic divergence was calculated based on the number of fixed differences between the reference and ancestral sequences, with polymorphic sites masked, relative to total region size.

### Calculating summary statistics from simulated data

We calculated summary statistics from simulated data in a manner that replicated the empirical data, using the same software as described above. Thus, sites that had been masked in the empirical data were also masked in the simulated data prior to calculating summary statistics.

Exonic divergence was calculated as the number of fixations post-burn-in from forward-in-time simulations (see the “*Simulating human population history with selection using SLiM*” section).

We calculated the mean and standard deviation for each region across its respective 100 replicates. For plotting purposes, we plotted the mean of all regions as the data point and the mean of the standard deviations across all regions as the confidence intervals.

### Simulating human population history using msprime

**Fig. 1.**
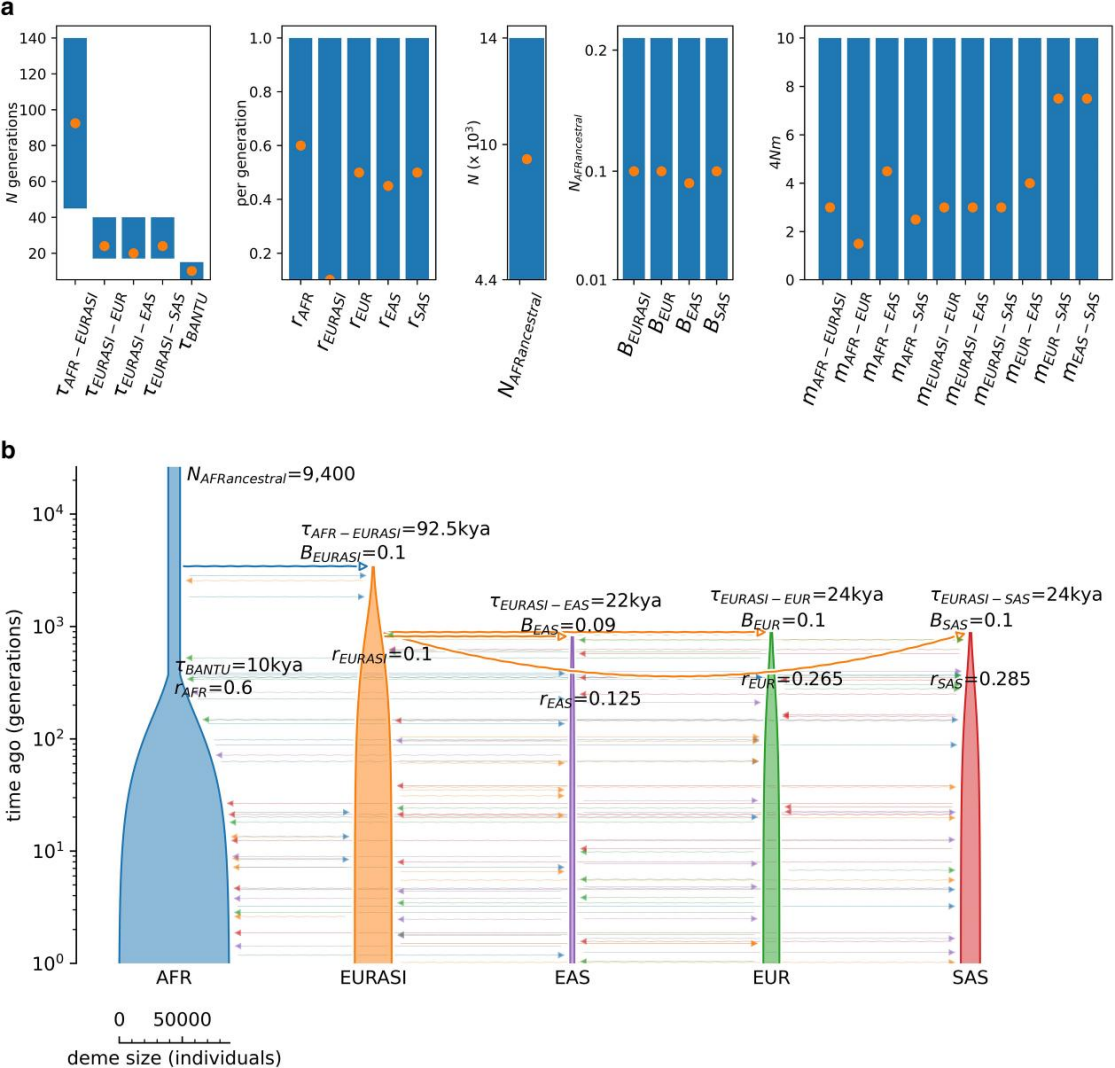
Demographic model representing the OOA expansion. a) Parameter ranges for all 25 parameters (represented by the blue bars on the plots). Orange dots indicate the best-fitting parameter values identified. b) Plot of demographic model with the best-fitting parameter values. Population key: AFRancestral, initial ancestral African population; AFR, African population; EURASI, unsampled Eurasian population; EUR, European population; EAS, East Asian population; SAS, South Asian population. Parameter key: *τ*, time of splits between specified populations (with *τ*_BANTU_ representing the time of start of the Bantu expansion in the African population); *r*, growth parameter; *N*, population size; *B*, bottleneck severity; *m*, migration rate. Demographic model graphic generated using Demes software (Gower *et al*. 2022).

Step 1 in our 2-step inference framework was the inference of population history. We simulated human demography using the coalescent simulator msprime (Baumdicker *et al*. 2022) for each of our 146 nonfunctional regions, with region-specific mutation and recombination rates, for each parameter combination. Our demographic model was comprised of 5 populations (4 sampled populations: African, European, South Asian, and East Asian; as well as the unsampled ancestral Eurasian population) and 25 parameters. Parameter ranges were taken from the human demographic inference literature, with midpoints of all ranges used as the initial starting parameterization. A generation time of 26.9 years was used to appropriately scale simulations (Wang *et al*. 2023). For details of the demographic model, see Fig. 1 and Supplementary Table 2. One hundred replicates were simulated for each of the 146 nonfunctional regions, with a single mutation and recombination rate per region, calculated as the average across the region from the Francioli *et al*. (2015) mutation rate map and the Halldorsson *et al*. (2019) recombination rate map (see Supplementary Fig. 1 for distributions of region lengths, mutation rates, and recombination rates across curated regions). Parameters were optimized to the data using *F*_ST_, the number of segregating sites, Tajima's *D* (Tajima 1989), and mean *r*^2^, across all 4 populations. Optimization was performed via an initially coarse grid search, followed by a finer-scaled grid search to further interrogate the high-likelihood parameter space. Demographic inference plots (e.g. Fig. 1) were produced using Demes software (Gower *et al*. 2022).

### Simulating human population history with selection using SLiM

For step 2, we simulated the inferred population history from step 1 using the forward-in-time simulator SLiM [v4.0.1 (Haller and Messer 2023)] for our 397 exonic regions, with region-specific mutation and recombination rates. We simulated to the human–chimpanzee split (12 mya; Moorjani *et al*. 2016). Thus, the simulations considered 12 million years (446,100 generations) before starting the 10*N* generation burn-in period. Exonic mutations were drawn from a DFE comprised of 4 fixed classes (following Johri *et al*. 2020), with frequencies denoted by *f_i_*: *f*_0_ with 0 ≤ 2*N*_AFRancestral_*s* < 1 (i.e. effectively neutral mutations), *f*_1_ with *1* ≤ 2*N*_AFRancestral_*s* < 10 (i.e. weakly deleterious mutations), *f*_2_ with 10 ≤ 2*N*_AFRancestral_*s* < 100 (i.e. moderately deleterious mutations), and *f*_3_ with 100 ≤ 2*N*_AFRancestral_*s* (i.e. strongly deleterious mutations), where 2*N*_AFRancestral_ is the initial African population size and *s* is the reduction in fitness of the mutant homozygote relative to the wild type. We initially simulated the DFE from Johri *et al*. (2023)—comprised of neutral and deleterious mutations—which fit the empirical data well.

### Simulating selective sweeps

#### Recurrent

We simulated recurrent selective sweeps by adding a beneficial DFE category for our 397 exonic regions. We simulated 3 different beneficial rates (0.1, 1, and 10% of new mutations), with the effectively neutral DFE category (*f*_0_) reduced to account for the addition of the beneficial category. Three different beneficial classes were separately simulated: 1 ≤ 2*N*_AFRancestral_*s_b_* < 10; 10 ≤ 2*N*_AFRancestral_*s_b_* < 100; and 100 ≤ 2*N*_AFRancestral_*s_b_* < 1,000, where *s_b_* is the increase in mutant homozygote fitness relative to the wild type.

#### Individual

To simulate a single hard selective sweep, we ran our inferred demographic model with the inferred DFE, with 3 different scenarios for introducing a beneficial mutation: model 1—the beneficial mutation was introduced into the African population immediately after burn-in; model 2—the beneficial mutation was introduced into the ancestral Eurasian population immediately after splitting from the African population; and model 3—the beneficial mutation was introduced into the European population immediately after splitting from the Eurasian population. In model 1, simulations were terminated and restarted if the beneficial mutation did not fix in all 4 sampled populations. In model 2, simulations were terminated and restarted if the hard sweep did not fix in the European, East Asian, and South Asian populations. Finally, in model 3, simulations were terminated and restarted if the hard sweep did not fix in the European population. For each scenario, two different strengths of selection were simulated: 2*N*_e_*s_b_* = 1,000 and 10,000, where *N*_e_ is the ancestral African population size (*N*_AFRancestral_) and *s_b_* is the beneficial selection coefficient.

For these simulations, we utilized the chromosomal structure of Soni and Jensen (2024), with functional regions comprised of 9 exons (each of size 1,317 bp) and 8 introns (each of size 1,520 bp), separated by intergenic regions (each of size 4,322 bp) (The 1000 Genomes Project Consortium 2015). The number of exons and introns per functional region was taken from Sakharkar *et al*. (2004). The chromosomal region contained 7 functional regions in total, resulting in a total simulated region length of 198,345 bp.

Variable mutation and recombination rates were drawn from a uniform distribution such that the mean recombination rate across the simulated region for each replicate was equal to the Kong *et al*. (2010) mean, and the mean mutation rate across the simulated region for each replicate was equal to the Kessler *et al*. (2020) mean.

### Sweep inference with SweepFinder2

We performed selective sweep inference by running SweepFinder2 (DeGiorgio *et al*. 2016) on each simulated replicate of each exonic region from our hard sweep simulations. Allele frequency files were generated for each replicate, following Huber *et al*.'s (2016) recommendation of including only polymorphic and substitution data. Inference was performed at each SNP via a grid file, following Nielsen *et al*. (2005). The background SFS was taken from the sweep-free simulations inferred in this study. The following command line was used for inference:

SweepFinder2 -lru GridFile FreqFile SpectFile RecFile OutFile.

### Sweep inference with H12

We ran the H12 method of Garud *et al*. (2015) on each simulated replicate of each exonic region from our hard sweep simulations, using a custom python script. H12 was estimated over 1 kb, 2 kb, 5 kb, 10 kb, 20 kb, and 40 kb windows at each SNP, with the SNP at the center of each window.

For both SweepFinder2 and H12 inference, we calculated true-positive rate (TPR) and false-positive rate (FPR) based on the inference values at each site, generating receiver operating characteristic (ROC) curves from this information.

## Results and discussion

Our implemented 2-step approach to demographic and DFE inference involves inferring population history using nonfunctional regions that are at a sufficient distance from functional sites so as to reasonably ensure that they are not experiencing purifying or BGS effects. DFE inference is then performed on exonic regions in step 2, conditional on the demographic history inferred in step 1 and incorporating expected BGS effects. We have applied this approach to human population genomic data from the 1000 Genomes Project (The 1000 Genomes Project Consortium 2015), in order to better characterize the evolutionary parameters governing recent human history.

### Step 1: demographic inference on nonfunctional regions

In order to avoid the biasing effects of purifying selection and BGS, we performed demographic inference on our curated set of 146 nonfunctional regions, with mean recombination and mutation rates calculated for each region from the rate maps of Halldorsson *et al*. (2019) and Francioli *et al*. (2015), respectively. For details of the data curation steps, please see the “*Materials and methods*” section. While one would typically begin with an evaluation of numerous demographic models and topologies in less well-characterized species (see Beaumont *et al*. 2002; Johri *et al*. 2020), given the considerable literature on human demographic history (e.g. Gutenkunst *et al*. 2009; Gravel *et al*. 2011; Schiffels and Durbin 2014; Terhorst *et al*. 2017; Hu *et al*. 2023), and inferred levels of admixture in The 1000 Genomes data set (The 1000 Genomes Project Consortium 2015), we began with a model of the OOA colonization in which the ancestral Eurasian population splits from the African population, followed by the European, South Asian, and East Asian populations dispersing from the ancestral Eurasian population, along with the Bantu expansion in the African population. Thus, our demographic model was comprised of 5 populations (African, ancestral Eurasian, European, South Asian, and East Asian, of which all but the ancestral Eurasian population were sampled) and 25 parameters that capture population sizes, bottleneck severities, growth rates, timings of each event, and migration rates between populations. Parameter ranges were drawn from the extensive literature on human population history (Mellars 2006; Gutenkunst *et al*. 2009; Gravel *et al*. 2011; Tennessen *et al*. 2012; Terhorst *et al*. 2017). Figure 1a provides the parameter ranges for our model, and see the “*Materials and methods*” section for further details.

**Fig. 2.**
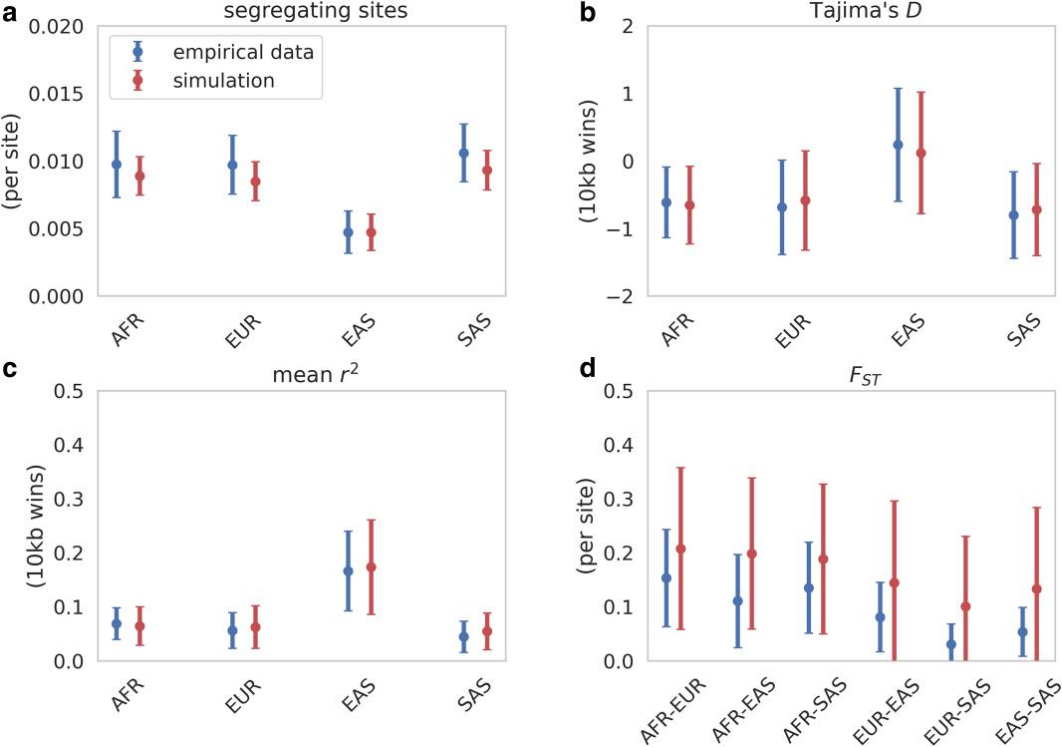
Summary statistics calculated from putatively neutral nonfunctional regions from population samples for empirical (blue) data, compared to simulated (red) data under the best-fitting demographic model. a) Number of segregating sites (calculated per site); b) Tajima's *D* (calculated across 10 kb sliding windows, with a 5 kb step size); c) mean *r*^2^ (calculated across 10 kb sliding windows, with a 5 kb step size); d) *F*_*ST*_ (calculated per site). Means and standard deviations were calculated for 100 replicates. Data points represent the mean across regions, while bars represent the mean of the standard deviations across all regions.

We simulated 100 replicates for each of our 146 nonfunctional regions using the coalescent simulator msprime (Baumdicker *et al*. 2022) with region-specific mutation and recombination rates, initially starting with midpoint values for each of our parameters (see Fig. 1a). For each replicate, we estimated 4 summary statistics for each population (or pairs of populations): the number of segregating sites, Tajima's *D* (Tajima 1989), mean *r*^2^, and *F*_ST_, giving us a total of 18 summary statistics. Fitting these 4 statistics via grid search enabled us to account for multiple aspects of the data including levels of diversity, the SFS, LD, and population structure. Figure 1a provides the optimized fit of each parameter within the context of previously published parameter ranges, and Fig. 1b the total inferred demographic model. As shown in Fig. 2, the summary statistics resulting from this demographic model well fit observed empirical data.

It is notable that the African population in our model is larger than the African populations in the Gutenkunst *et al*. (2009) and Gravel *et al*. (2011) best-fitting models. There are 2 likely contributing factors. Firstly, these previous studies fit the model to the SFS, whereas we have here fit additional summaries of the data. Secondly, these previous studies modeled the African population with a fixed size that undergoes a single instantaneous expansion. Here we modeled the recent Bantu expansion, and thus, our final African population size was notably larger, though the size of 87,594 falls within the range of previous estimates (Schiffels and Durbin 2014; Terhorst *et al*. 2017; Johri *et al*. 2023). Finally, it is worth noting that numerous other coalescent and diffusion approximation-based approaches have been used to infer the OOA model of human population history (Gutenkunst *et al*. 2009; Gravel *et al*. 2011; Excoffier *et al*. 2013; Jouganous *et al*. 2017; Wang *et al*. 2020). These studies have masked genic regions to avoid the biasing effects of selection. However, BGS can still affect demographic inference if not accounted for; nonetheless, our parameter estimates fall within previously inferred ranges, confirming the modest nature of BGS effects in humans (Johri *et al*. 2021; Buffalo and Kern 2024).

In summary, by optimizing within previously published parameter ranges, we have identified a neutral demographic model that well explains multiple facets of the genomic data in distant noncoding regions.

### Step 2: DFE inference on functional regions

**Fig. 3.**
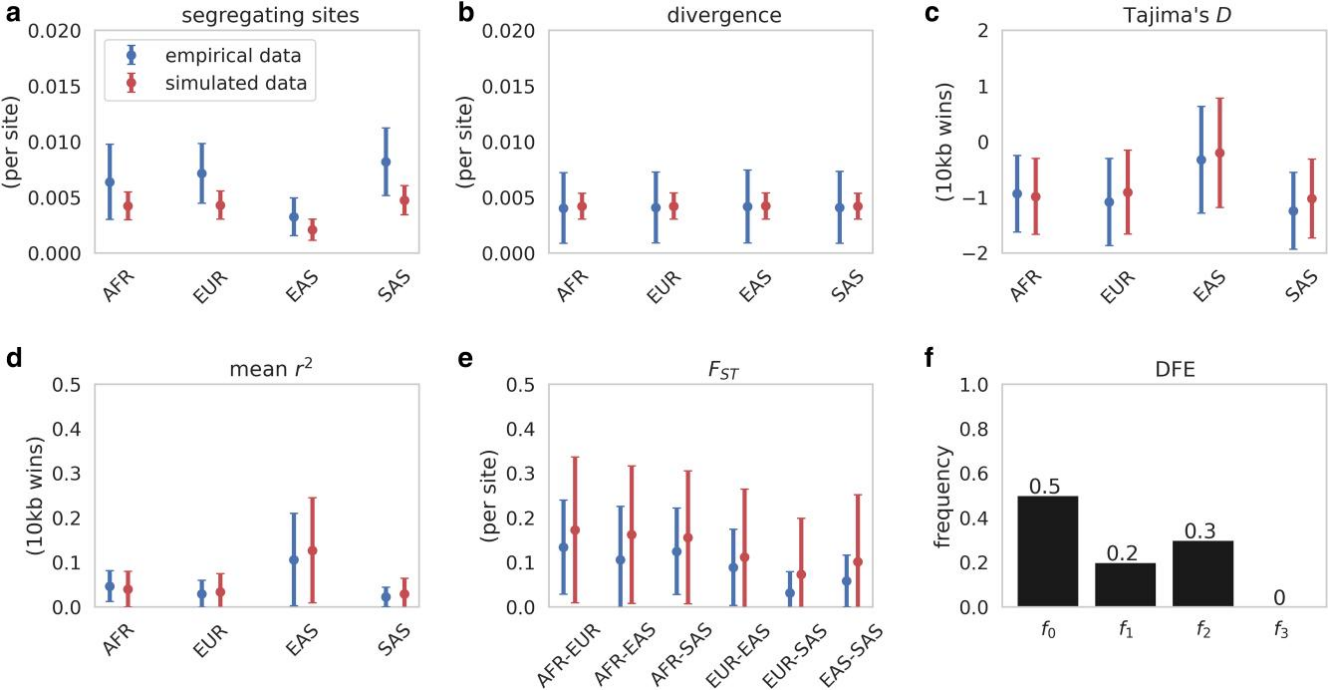
a to e) Summary statistics calculated from functional regions from population samples for empirical (blue) data, compared to simulated (red) data under the best-fitting neutral demographic model with the addition of purifying and BGS modeled using the Johri *et al*. (2023) DFE (shown in f). Following this DFE, exonic mutations were drawn from a DFE comprised of 4 fixed classes with frequencies denoted by *f_i_*: *f*_0_ with 0 ≤ 2*N*_AFRancestral_*s* < 1 (i.e. effectively neutral mutations), *f*_1_ with 1 ≤ 2*N*_AFRancestral_*s* < 10 (i.e. weakly deleterious mutations), *f*_2_ with 10 ≤ 2*N*_AFRancestral_*s* < 100 (i.e. moderately deleterious mutations), and *f*_3_ with 100 ≤ 2*N*_AFRancestral_*s* (i.e. strongly deleterious mutations), where *N*_AFRancestral_ was the initial population size and *s* the reduction in fitness of the mutant homozygote relative to wild type. Means and standard deviations were calculated for 100 replicates. Data points represent the mean across regions, while bars represent the mean of the standard deviations across all regions.

Given the strong fit of the neutral demographic model to the intergenic data, we next moved to step 2, inference of the DFE using functional regions. We utilized the curated set of functional regions from Johri *et al*. (2023). After obtaining region-specific mutation and recombination rates, we were left with a total of 397 functional regions. Unlike Johri *et al*. (2023) who simulated exons and their neighboring regions, we focused on the exons only (given that the model fit was consistent across both exons and adjacent regions in their study). First, we simulated our 397 functional regions under the demographic model inferred in step 1, using the forward-in-time simulator SLiM [v4.0.1 (Haller and Messer 2023)]. For the purpose of DFE inference, we simulated to the human–chimpanzee split time [12 mya (Moorjani *et al*. 2016)] to allow us to compare empirical and simulated divergence, which is expected to be shaped by selection at functional sites. When simulating these functional regions under selective neutrality, we found that the fit to the empirical data was poorer than for the nonfunctional regions (Supplementary Fig. 2): an expected result given the action of selection in these exonic regions. Next, we simulated under the Johri *et al*. (2023) DFE using our fit demographic model and found a good fit of the simulated summary statistics to the empirical data (Fig. 3). These results are encouraging given the differing approaches taken between the 2 studies: we here took the 2-step approach as described, while Johri *et al*. utilized a simultaneous inference scheme. Importantly however, both studies accounted for expected BGS effects, a relative rarity in DFE inference.

Though the inclusion of population history, purifying and BGS effects, and mutation and recombination rate heterogeneity were alone sufficient to explain empirically observed data patterns, that does not necessarily imply the absence of beneficial mutations; rather, it suggests that this additional parameter is not needed in order to fit observed patterns of variation. While this observation is itself meaningful, it indeed raises the question of what rate of beneficial mutation may be consistent with the data but simply unidentifiable. In order to investigate this question, we added a beneficial DFE category to the Johri *et al*. (2023) DFE, in an attempt to understand what rate of input of beneficial mutations may be compatible with the observed levels of variation, the SFS, LD, divergence, and *F*_ST_. Initially, we considered 3 beneficial DFE proportions, *f_bo_* = [0.1, 1, or 10% of newly arising mutations], with 1 ≤ 2*N*_AFRancestral_*s_b_* < 10 (i.e. weakly beneficial mutations). Under this model, we correspondingly reduced *f*_0_—the proportion of effectively neutral mutations—in order to account for the addition of this beneficial DFE class. Supplementary Figs. 3–5 provide the fit of the summary statistics from these simulations to the observed data. At *f_b_*_0_ = 0.1 or 1%, all summary statistics remain reasonably well fit—in other words, they are not significantly modified from the expectations in the absence of positive selection. However, divergence was notably increased relative to that observed at *f_b_*_0_ = 10%, due to the greater rate of beneficial fixation.

Given that this beneficial mutation rate of 10% appears inconsistent with empirical divergence, we next examined *f_b_*_0_ = 0.1 and 1% only while increasing the population-scaled strength of selection to 10 ≤ 2*N*_AFRancestral_*s_b_* < 100 (i.e. moderately beneficial mutations). Supplementary Figs. 6 and 7 provide the fit of summary statistics from these simulations to the observed data. With this increased strength of selection, the modeled divergence only fit the empirical data at the lowest beneficial frequency, *f_B_*_0_ = 0.1%. Finally, we increased the population-scaled strength of selection further to 100 ≤ 2*N*_AFRancestral_*s_b_* < 1,000 (i.e. strongly beneficial mutations), at *f_B_*_0_ = 0.1%. Even at this low frequency, the resulting divergence was too high relative to the empirical data (see Supplementary Fig. 8 for all summary statistics). It is notable that regardless of beneficial mutation frequency or strength of selection, the other summary statistics fit the data well—this owes to the relative waiting time between selective sweeps under these models; that is, selective sweeps are too old on average to strongly impact patterns of polymorphism (Jensen 2009) while being frequent enough to modify divergence over the 12 mya timescale.

Though the ability to estimate a beneficial DFE category independently remains to be examined, these results suggest that while the addition of a beneficial DFE class is not necessary to explain the patterns observed in the human population genomic data here considered, a modest input of weakly beneficial mutations and/or a low input of moderately beneficial mutations would remain consistent with the observed data. However, a coarse DFE consisting only of neutral, weakly deleterious, and moderately deleterious mutations is sufficient to well-fit the observed data.

### Evaluating power to detect selective sweeps within this human baseline model

Recurrent sweep models, such as the one studied above, involve a scenario in which beneficial mutations occur randomly across a chromosome according to a time-homogenous Poisson process at a per-generation rate (Kaplan *et al*. 1989; Wiehe and Stephan 1993; Stephan 1995; Pavlidis *et al*. 2010; Soni *et al*. 2023). Although this is a more realistic model of positive selection, in that the beneficial mutations underlying selective sweeps naturally occur at a per-generation rate—meaning that they are naturally associated with an average time since fixation—the more commonly studied model involves a single selective sweep in which fixation occurred immediately prior to sampling. As such, these models consider a best-case scenario for sweep detection, both in that sweeps are as recent as possible thus maximizing detectable polymorphism-based patterns (see review of Nielsen 2005), but also because it avoids the possibility of interference between positively selected mutations (i.e. Hill and Robertson 1966).

Furthermore, these models are often simulated on otherwise neutral backgrounds, which is additionally unrealistic in the sense that beneficial mutations occur in functional regions, which will be dominated by newly arising deleterious mutations. Thus, as a step toward biological reality, we here have modeled single selective sweeps within the context of our evolutionary baseline model, using our inferred demographic history, DFE, as well as mutation and recombination rate maps, thereby accounting for constantly operating evolutionary processes in order to characterize the power to identify an episodic selective sweep (as described by Johri, Aquadro, *et al*. 2022).

**Fig. 4.**
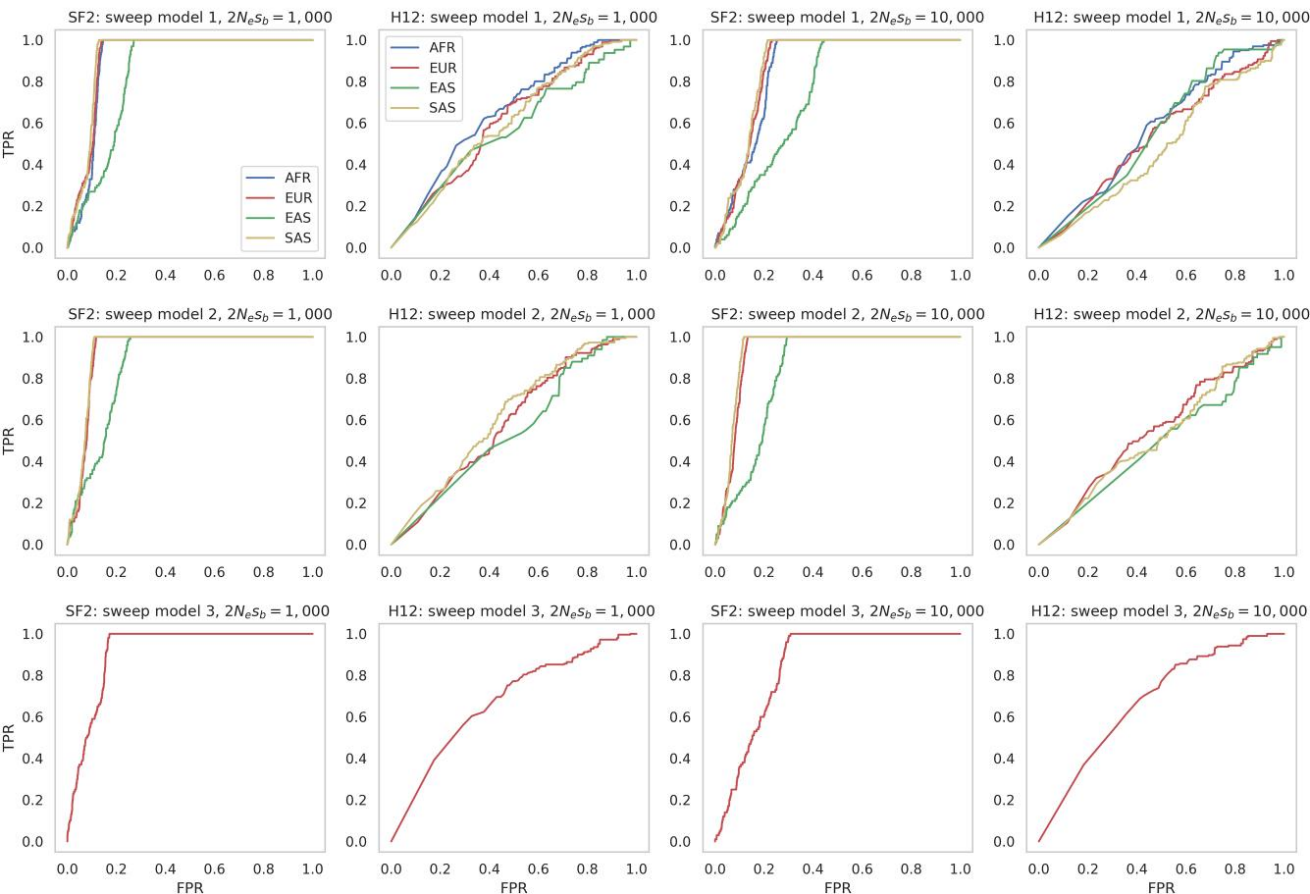
ROC curves, providing the change in TPR with FPR, for sweep inference with SweepFinder2 (SF2) and the H12 statistic under the demographic model inferred in this study (see Fig. 1) together with the Johri *et al*. (2023) DFE for functional regions, and variable mutation and recombination rates (see the “*Materials and methods*” section). Here, a single beneficial mutation was introduced into the population at 3 different time points and in 3 different populations: model 1—the beneficial mutation was introduced into the ancestral African population immediately after the burn-in period, the beneficial fixation is present in all populations, and sweep inference was conducted on all sampled populations; model 2—the beneficial mutation was introduced into the ancestral Eurasian population immediately upon splitting from the ancestral African population, the beneficial fixation is present in all non-African populations, and sweep inference was conducted on the European, East Asian, and South Asian populations; model 3—the beneficial mutation was introduced into the European population immediately upon splitting from the Eurasian population, the beneficial fixation is present in the European population, and sweep inference was conducted on this population only. For each model, 2 different strengths of selection were modeled: 2*N*_e_*s_b_* = 1,000 and 2*N*_e_*s_b_* = 10,000, where *N*_e_ is the size of the ancestral African population and *s_b_* is the selection coefficient of the beneficial mutation. Inference with SweepFinder2 was performed on each SNP and substitution, while H12 inference was performed on each SNP over a 1 kb window, with the SNP at the center of the window.

Under this model, we simulated a large genomic region comprised of functional and nonfunctional regions in which a single hard selective sweep occurred in a functional element (see the “*Materials and methods*” section for more details about simulated chromosomal structure, as well as parameterizations). Sweep inference was conducted using two methods: the composite likelihood ratio SFS-based method, SweepFinder2 (DeGiorgio *et al*. 2016), and a haplotype-based approach, H12. Three different sweep models were simulated: (1) a beneficial mutation introduced into the ancestral African population immediately after simulation burn-in, with the fixed beneficial present in the sampled African, European, East Asian, and South Asian populations; (2) a beneficial mutation introduced into the ancestral Eurasian population immediately after splitting from the ancestral African population, with the fixed beneficial present in the sampled European, East Asian, and South Asian populations; and (3) a beneficial mutation introduced into the European population immediately after splitting from the Eurasian population, with the fixed beneficial present in the sampled European population. Figure 4 presents ROC plots, plotting the FPR against the TPR for inference on each model across 100 replicates with SweepFinder2 (with inference performed at each SNP) and H12 (with inference performed across 1 kb windows, centered on each SNP; see Supplementary Figs. 9–13 for additional window sizes).

At the lowest strength of selection (2*N*_e_*s_b_* = 100), no beneficial mutations reached fixation by the sampling time (i.e. the present day) across the replicates. As such, Fig. 4 presents ROC plots for 2*N*_e_*s_b_* values of 1,000 and 10,000 only. Although SweepFinder2 showed greater inference power than H12, there was limited power to detect selective sweeps for both approaches. While potentially appearing counter intuitive, in some circumstances, 2*N*_e_*s_b_* = 1,000 had greater power than 2*N*_e_*s_b_* = 10,000, as the fixations of the former were more recent given the longer sojourn time, and thus experienced less postfixation decay in patterns of polymorphism (Kim and Stephan 2000; Soni *et al*. 2023). These results thus suggest that detectable selective sweeps would necessarily be the result of positive selection that was strong and recent enough to leave a detectable signature, consistent with previous work (Kim and Stephan 2002; Przeworski 2002; Jensen *et al*. 2007; Crisci *et al*. 2013). Moreover, the modest power under our baseline model is likely explained by the severe bottlenecks and expansions characterizing these populations, as the fundamental difficulty in distinguishing between population bottlenecks and selective sweeps has been previously demonstrated (Barton 1998; Jensen *et al*. 2005). These results suggest that caution is needed when performing genomic scans for selection in humans owing to their complex demographic history. Furthermore, these results lend credence to previous assertions that strong selective sweeps may have been rare in recent human history (Hernandez *et al*. 2011).

### Conclusion

In this study, we have demonstrated the viability of a 2-step approach for inferring population history along with the DFE in coding-sparse genomes, such as that characterizing humans. This condition, together with being a recombining genome, is important for the existence and availability of nonfunctional regions sufficiently distant from functional sites so as to be free from the effects of purifying and BGS, as such regions are necessary for the accurate inference of population history. By contrast, organisms with genomes that are either coding dense or experience limited recombination may not have such regions in sufficient number, in which case demographic inference must be performed within the context of BGS effects. As these BGS effects will be dictated partially by the DFE in functional regions, this genomic architecture requires the joint and simultaneous inference of demographic and selective parameters—a situation that spans organisms ranging from *Drosophila* to many viruses (see review of Johri, Eyre-Walker, *et al*. 2022). However, given the multiple jointly inferred parameters, the demographic histories under these joint inference schemes have been highly simplified in current implementations. Thus, this 2-step approach has a distinct advantage for coding-sparse genomes, in that previously developed and sophisticated neutral demographic inference approaches may be leveraged in step 1—such as that employed here estimating a 25-parameter human demographic model consisting of multiple population size changes, split times, and migration rates—allowing DFE inference to be focused upon in step 2 conditional on that inferred history. SFS-based likelihood frameworks [e.g. fastsimcoal2 (Excoffier *et al*. 2013) or *δαδi* (Gutenkunst *et al*. 2009)] may equally be used for demographic inference in step 1, though ABC approaches have the benefit of fitting multiple aspects of the data in addition to the SFS alone. The computational demands of ABC approaches will depend on the nature of the data itself, in terms of number of populations, underlying recombination rate maps, and so on. Within the context of likelihood-based approaches, these demographic parameters are associated with confidence intervals, which can be sampled from when performing DFE inference, in order to account for underlying uncertainty. Within the context of approximate Bayesian-based approaches, these uncertainties are captured in the posterior distributions and associated credibility intervals, which can similarly be sampled from when performing downstream inference.

It is additionally important to consider the extent to which a consideration of these BGS effects matters for human demographic inference. Indeed, given the coding sparseness of the genome, these effects are expected a priori to be limited, and that is fully consistent with the observation that our optimized demographic parameter values fall within previously published parameter ranges. However, apart from accounting for the effects of selection at linked sites, this approach also utilizes patterns of variation in addition to the SFS (e.g. LD and population differentiation), which provide a further valuable “sanity check” on estimated models. This combination of factors has resulted in incrementally improved—but indeed improved—parameter estimates for the populations studied, as assessed by the fit between the estimated model and the empirical data. Thus, this proof-of-principle approach applied here to publicly available human data will likely provide a highly relevant and informative inference framework for the analysis of future genomic resources in comparatively poorly studied species with a similar genomic architecture (e.g. nonhuman primates).

## Supplementary Material

jkaf019_Supplementary_Data

## Data Availability

Code to run simulations and perform analyses is available on GitHub: (https://github.com/vivaksoni/human_demog_DFE/). Supplemental material available at G3 online.

## References

[jkaf019-B1] Bank C , Ewing GB, Ferrer-Admettla A, Foll M, Jensen JD. 2014. Thinking too positive? Revisiting current methods of population genetic selection inference. Trends Genet. 30(12):540–546. doi:10.1016/j.tig.2014.09.010. 25438719

[jkaf019-B2] Barton NH . 1998. The effect of hitch-hiking on neutral genealogies. Genet Res. 72(2):123–133. doi:10.1017/S0016672398003462.

[jkaf019-B3] Baumdicker F , Bisschop G, Goldstein D, Gower G, Ragsdale AP, Tsambos G, Zhu S, Eldon B, Ellerman EC, Galloway JG, et al 2022. Efficient ancestry and mutation simulation with msprime 1.0. Genetics. 220(3):iyab229. doi:10.1093/genetics/iyab229. 34897427 PMC9176297

[jkaf019-B4] Beaumont MA , Zhang W, Balding DJ. 2002. Approximate Bayesian computation in population genetics. Genetics. 162(4):2025–2035. doi:10.1093/genetics/162.4.2025. 12524368 PMC1462356

[jkaf019-B5] Boyko AR , Williamson SH, Indap AR, Degenhardt JD, Hernandez RD, Lohmueller KE, Adams MD, Schmidt S, Sninsky JJ, Sunyaev SR, et al 2008. Assessing the evolutionary impact of amino acid mutations in the human genome. PLoS Genet. 4(5):e1000083. doi:10.1371/journal.pgen.1000083. 18516229 PMC2377339

[jkaf019-B6] Buffalo V , Kern AD. 2024. A quantitative genetic model of background selection in humans. PLoS Genet. 20(3):e1011144. doi:10.1371/journal.pgen.1011144. 38507461 PMC10984650

[jkaf019-B7] Charlesworth B , Jensen JD. 2021. Effects of selection at linked sites on patterns of genetic variability. Annu Rev Ecol Evol Syst. 52(1):177–197. doi:10.1146/annurev-ecolsys-010621-044528. 37089401 PMC10120885

[jkaf019-B8] Charlesworth B , Jensen JD. 2022. Some complexities in interpreting apparent effects of hitchhiking: a commentary on Gompert et al. (2022). Mol Ecol. 31(17):4440–4443. doi:10.1111/mec.16573. 35778972 PMC9536517

[jkaf019-B9] Charlesworth B , Jensen JD. 2024. Population genetics. Encyclopedia of Biodiversity. 3rd ed. Elsevier Ltd. Vol. 7, p. 467–483.

[jkaf019-B10] Charlesworth B , Morgan MT, Charlesworth D. 1993. The effect of deleterious mutations on neutral molecular variation. Genetics. 134(4):1289–1303. doi:10.1093/genetics/134.4.1289. 8375663 PMC1205596

[jkaf019-B11] Crisci JL , Poh YP, Mahajan S, Jensen JD. 2013. The impact of equilibrium assumptions on tests of selection. Front Genet. 4:235. doi:10.3389/fgene.2013.00235. 24273554 PMC3822286

[jkaf019-B12] Crow JF . 1987. Muller, Dobzhansky, and overdominance. J Hist Biol. 20(3):351–380. doi:10.1007/BF00139460.

[jkaf019-B13] Cunningham F , Allen JE, Allen J, Alvarez-Jarreta J, Amode MR, Armean IM, Austine-Orimoloye O, Azov AG, Barnes I, Bennett R, et al 2022. Ensembl 2022. Nucleic Acids Res. 50(D1):D988–D995. doi:10.1093/nar/gkab1049. 34791404 PMC8728283

[jkaf019-B14] Cutter AD , Payseur BA. 2013. Genomic signatures of selection at linked sites: unifying the disparity among species. Nat Rev Genet. 14(4):262–274. doi:10.1038/nrg3425. 23478346 PMC4066956

[jkaf019-B15] DeGiorgio M , Huber CD, Hubisz MJ, Hellmann I, Nielsen R. 2016. SweepFinder2: increased sensitivity, robustness, and flexibility. Bioinformatics. 32(12):1895–1897. doi:10.1093/bioinformatics/btw051. 27153702

[jkaf019-B16] Dobzhansky T . 1955. A review of some fundamental concepts and problems of population genetics. Cold Spring Harb Symp Quant Biol. 20(0):1–15. doi:10.1101/SQB.1955.020.01.003. 13433550

[jkaf019-B17] Ewing GB , Jensen JD. 2014. Distinguishing neutral from deleterious mutations in growing populations. Front Genet. 5:7. doi:10.3389/fgene.2014.00007. 24550931 PMC3907712

[jkaf019-B18] Ewing GB , Jensen JD. 2016. The consequences of not accounting for background selection in demographic inference. Mol Ecol. 25(1):135–141. doi:10.1111/mec.13390. 26394805

[jkaf019-B19] Excoffier L , Dupanloup I, Huerta-Sánchez E, Sousa VC, Foll M. 2013. Robust demographic inference from genomic and SNP data. PLoS Genet. 9(10):e1003905. doi:10.1371/journal.pgen.1003905. 24204310 PMC3812088

[jkaf019-B20] Eyre-Walker A , Keightley PD. 2007. The distribution of fitness effects of new mutations. Nat Rev Genet. 8(8):610–618. doi:10.1038/nrg2146. 17637733

[jkaf019-B21] Eyre-Walker A , Keightley PD. 2009. Estimating the rate of adaptive molecular evolution in the presence of slightly deleterious mutations and population size change. Mol Biol Evol. 26(9):2097–2108. doi:10.1093/molbev/msp119. 19535738

[jkaf019-B22] Flicek P , Amode MR, Barrell D, Beal K, Billis K, Brent S, Carvalho-Silva D, Clapham P, Coates G, Fitzgerald S, et al 2014. Ensembl 2014. Nucleic Acids Res. 42(Database issue):D749–D755. doi:10.1093/nar/gkt1196. 24316576 PMC3964975

[jkaf019-B23] Francioli LC , Polak PP, Koren A, Menelaou A, Chun S, Renkens I, van Duijn CM, Swertz M, Wijmenga C, van Ommen G, et al 2015. Genome-wide patterns and properties of de novo mutations in humans. Nat Genet. 47(7):822–826. doi:10.1038/ng.3292. 25985141 PMC4485564

[jkaf019-B24] Galtier N . 2016. Adaptive protein evolution in animals and the effective population size hypothesis. PLoS Genet. 12(1):e1005774. doi:10.1371/journal.pgen.1005774. 26752180 PMC4709115

[jkaf019-B25] Garud NR , Messer PW, Buzbas EO, Petrov DA. 2015. Recent selective sweeps in North American *Drosophila melanogaster* show signatures of soft sweeps. PLoS Genet. 11(2):e1005004. doi:10.1371/journal.pgen.1005004. 25706129 PMC4338236

[jkaf019-B26] Gower G , Ragsdale AP, Bisschop G, Gutenkunst RN, Hartfield M, Noskova E, Schiffels S, Struck TJ, Kelleher J, Thornton KR. 2022. Demes: a standard format for demographic models. Genetics. 222(3):iyac131. doi:10.1093/genetics/iyac131. 36173327 PMC9630982

[jkaf019-B27] Gravel S , Henn BM, Gutenkunst RN, Indap AR, Marth GT, Clark AG, Yu F, Gibbs RA; The 1000 Genomes Project; Bustamante CD. 2011. Demographic history and rare allele sharing among human populations. Proc Natl Acad Sci U S A. 108(29):11983–11988. doi:10.1073/pnas.1019276108. 21730125 PMC3142009

[jkaf019-B28] Gutenkunst RN , Hernandez RD, Williamson SH, Bustamante CD. 2009. Inferring the joint demographic history of multiple populations from multidimensional SNP frequency data. PLoS Genet. 5(10):e1000695. doi:10.1371/journal.pgen.1000695. 19851460 PMC2760211

[jkaf019-B29] Halldorsson BV , Palsson G, Stefansson OA, Jonsson H, Hardarson MT, Eggertsson HP, Gunnarsson B, Oddsson A, Halldorsson GH, Zink F, et al 2019. Characterizing mutagenic effects of recombination through a sequence-level genetic map. Science. 363(6425):eaau1043. doi:10.1126/science.aau1043. 30679340

[jkaf019-B30] Haller BC , Messer PW. 2023. SLiM 4: multispecies eco-evolutionary modeling. Am Nat. 201(5):E127–E139. doi:10.1086/723601. 37130229 PMC10793872

[jkaf019-B31] Harris RB , Jensen JD. 2020. Considering genomic scans for selection as coalescent model choice. Genome Biol Evol. 12(6):871–877. doi:10.1093/gbe/evaa093. 32396636 PMC7313662

[jkaf019-B33] Hernandez RD , Kelley JL, Elyashiv E, Melton SC, Auton A, McVean G; 1000 Genomes Project; Sella G, Przeworski M. 2011. Classic selective sweeps were rare in recent human evolution. Science. 331(6019):920–924. doi:10.1126/science.1198878. 21330547 PMC3669691

[jkaf019-B34] Hill WG , Robertson A. 1966. The effect of linkage on limits to artificial selection. Genet Res. 8(3):269–294. doi:10.1017/S0016672300010156. 5980116

[jkaf019-B35] Howell AA , Terbot J, Soni V, Johri P, Jensen JD, Pfeifer SP. 2023. Developing an appropriate evolutionary baseline model for the study of human cytomegalovirus. Genome Biol Evol. 15(4):evad059. doi:10.1093/gbe/evad059. 37071785 PMC10139446

[jkaf019-B36] Hu W , Hao Z, Du P, Di Vincenzo F, Manzi G, Cui J, Fu YX, Pan YH, Li H. 2023. Genomic inference of a severe human bottleneck during the Early to Middle Pleistocene transition. Science. 381(6661):979–984. doi:10.1126/science.abq7487. 37651513

[jkaf019-B37] Huang X , Fortier AL, Coffman AJ, Struck TJ, Irby MN, James JE, León-Burguete JE, Ragsdale AP, Gutenkunst RN. 2021. Inferring genome-wide correlations of mutation fitness effects between populations. Mol Biol Evol. 38(10):4588–4602. doi:10.1093/molbev/msab162. 34043790 PMC8476148

[jkaf019-B38] Huber CD , DeGiorgio M, Hellmann I, Nielsen R. 2016. Detecting recent selective sweeps while controlling for mutation rate and background selection. Mol Ecol. 25(1):142–156. doi:10.1111/mec.13351. 26290347 PMC5082542

[jkaf019-B39] Irwin KK , Laurent S, Matuszewski S, Vuilleumier S, Ormond L, Shim H, Bank C, Jensen JD. 2016. On the importance of skewed offspring distributions and background selection in virus population genetics. Heredity (Edinb). 117(6):393–399. doi:10.1038/hdy.2016.58. 27649621 PMC5117839

[jkaf019-B40] Jensen JD . 2009. On reconciling single and recurrent hitchhiking models. Genome Biol Evol. 1:320–324. doi:10.1093/gbe/evp031. 20333201 PMC2817426

[jkaf019-B41] Jensen JD . 2021. Studying population genetic processes in viruses: from drug-resistance evolution to patient infection dynamics. Encyclopedia of Virology. 5th ed. Elsevier. p. 227–232.

[jkaf019-B42] Jensen JD . 2023. Population genetic concerns related to the interpretation of empirical outliers and the neglect of common evolutionary processes. Heredity (Edinb). 130(3):109–110. doi:10.1038/s41437-022-00575-5. 36829044 PMC9981695

[jkaf019-B43] Jensen JD , Kim Y, DuMont VB, Aquadro CF, Bustamante CD. 2005. Distinguishing between selective sweeps and demography using DNA polymorphism data. Genetics. 170(3):1401–1410. doi:10.1534/genetics.104.038224. 15911584 PMC1451184

[jkaf019-B44] Jensen JD , Payseur BA, Stephan W, Aquadro CF, Lynch M, Charlesworth D, Charlesworth B. 2019. The importance of the neutral theory in 1968 and 50 years on: a response to Kern and Hahn 2018. Evolution. 73(1):111–114. doi:10.1111/evo.13650. 30460993 PMC6496948

[jkaf019-B45] Jensen JD , Thornton KR, Bustamante CD, Aquadro CF. 2007. On the utility of linkage disequilibrium as a statistic for identifying targets of positive selection in nonequilibrium populations. Genetics. 176(4):2371–2379. doi:10.1534/genetics.106.069450. 17565955 PMC1950638

[jkaf019-B46] Johri P , Aquadro CF, Beaumont M, Charlesworth B, Excoffier L, Eyre-Walker A, Keightley PD, Lynch M, McVean G, Payseur BA, et al 2022. Recommendations for improving statistical inference in population genomics. PLoS Biol. 20(5):e3001669. doi:10.1371/journal.pbio.3001669. 35639797 PMC9154105

[jkaf019-B47] Johri P , Charlesworth B, Jensen JD. 2020. Toward an evolutionarily appropriate null model: jointly inferring demography and purifying selection. Genetics. 215(1):173–192. doi:10.1534/genetics.119.303002. 32152045 PMC7198275

[jkaf019-B48] Johri P , Eyre-Walker A, Gutenkunst RN, Lohmueller KE, Jensen JD. 2022. On the prospect of achieving accurate joint estimation of selection with population history. Genome Biol Evol. 14(7):evac088. doi:10.1093/gbe/evac088. 35675379 PMC9254643

[jkaf019-B49] Johri P , Pfeifer SP, Jensen JD. 2023. Developing an evolutionary baseline model for humans: jointly inferring purifying selection with population history. Mol Biol Evol. 40(5):msad100. doi:10.1093/molbev/msad100. 37128989 PMC10195113

[jkaf019-B50] Johri P , Riall K, Becher H, Excoffier L, Charlesworth B, Jensen JD. 2021. The impact of purifying and background selection on the inference of population history: problems and prospects. Mol Biol Evol. 38(7):2986–3003. doi:10.1093/molbev/msab050. 33591322 PMC8233493

[jkaf019-B51] Jouganous J , Long W, Ragsdale AP, Gravel S. 2017. Inferring the joint demographic history of multiple populations: beyond the diffusion approximation. Genetics. 206(3):1549–1567. doi:10.1534/genetics.117.200493. 28495960 PMC5500150

[jkaf019-B52] Kaplan NL , Hudson RR, Langley CH. 1989. The ‘hitchhiking effect’ revisited. Genetics. 123(4):887–899. doi:10.1093/genetics/123.4.887. 2612899 PMC1203897

[jkaf019-B53] Karolchik D , Baertsch R, Diekhans M, Furey TS, Hinrichs A, Lu YT, Roskin KM, Schwartz M, Sugnet CW, Thomas DJ, et al 2003. The UCSC genome browser database. Nucleic Acids Res. 31(1):51–54. doi:10.1093/nar/gkg129. 12519945 PMC165576

[jkaf019-B54] Keightley PD , Eyre-Walker A. 2007. Joint inference of the distribution of fitness effects of deleterious mutations and population demography based on nucleotide polymorphism frequencies. Genetics. 177(4):2251–2261. doi:10.1534/genetics.107.080663. 18073430 PMC2219502

[jkaf019-B55] Kessler MD , Loesch DP, Perry JA, Heard-Costa NL, Taliun D, Cade BE, Wang H, Daya M, Ziniti J, Datta S, et al 2020. De novo mutations across 1,465 diverse genomes reveal mutational insights and reductions in the Amish founder population. Proc Natl Acad Sci U S A. 117(5):2560–2569. doi:10.1073/pnas.1902766117. 31964835 PMC7007577

[jkaf019-B56] Kim BY , Huber CD, Lohmueller KE. 2017. Inference of the distribution of selection coefficients for new nonsynonymous mutations using large samples. Genetics. 206(1):345–361. doi:10.1534/genetics.116.197145. 28249985 PMC5419480

[jkaf019-B57] Kim Y . 2006. Allele frequency distribution under recurrent selective sweeps. Genetics. 172(3):1967–1978. doi:10.1534/genetics.105.048447. 16361239 PMC1456267

[jkaf019-B58] Kim Y , Stephan W. 2000. Joint effects of genetic hitchhiking and background selection on neutral variation. Genetics. 155(3):1415–1427. doi:10.1093/genetics/155.3.1415. 10880499 PMC1461159

[jkaf019-B59] Kim Y , Stephan W. 2002. Detecting a local signature of genetic hitchhiking along a recombining chromosome. Genetics. 160(2):765–777. doi:10.1093/genetics/160.2.765. 11861577 PMC1461968

[jkaf019-B60] Kimura M . 1968. Evolutionary rate at the molecular level. Nature. 217(5129):624–626. doi:10.1038/217624a0. 5637732

[jkaf019-B61] Kimura M . 1983. The Neutral Theory of Molecular Evolution. Cambridge, UK: Cambridge University Press.

[jkaf019-B62] Kong A , Thorleifsson G, Gudbjartsson DF, Masson G, Sigurdsson A, Jonasdottir A, Walters GB, Jonasdottir A, Gylfason A, Kristinsson KT, et al 2010. Fine-scale recombination rate differences between sexes, populations, and individuals. Nature. 467(7319):1099–1103. doi:10.1038/nature09525. 20981099

[jkaf019-B63] Kousathanas A , Keightley PD. 2013. A comparison of models to infer the distribution of fitness effects of new mutations. Genetics. 193(4):1197–1208. doi:10.1534/genetics.112.148023. 23341416 PMC3606097

[jkaf019-B64] Lewontin RC . 1987. Polymorphism and heterosis: old wine in new bottles and vice versa. J Hist Biol. 20(3):337–349. doi:10.1007/BF00139459.

[jkaf019-B65] Li H , Stephan W. 2006. Inferring the demographic history and rate of adaptive substitution in Drosophila. PLoS Genet. 2(10):e166. doi:10.1371/journal.pgen.0020166. 17040129 PMC1599771

[jkaf019-B66] Matuszewski M , Hildebrandt M, Achaz G, Jensen JD. 2018. Coalescent processes with skewed offspring distributions and non-equilibrium demography. Genetics. 208(1):323–338. doi:10.1534/genetics.117.300499. 29127263 PMC5753866

[jkaf019-B67] Maynard Smith J , Haigh J. 1974. The hitch-hiking effect of a favourable gene. Genet Res. 23(1):23–35. doi:10.1017/S0016672300014634. 4407212

[jkaf019-B68] McVicker G , Gordon D, Davis C, Green P. 2009. Widespread genomic signatures of natural selection in hominid evolution. PLoS Genet. 5(5):e1000471. doi:10.1371/journal.pgen.1000471. 19424416 PMC2669884

[jkaf019-B69] Mellars P . 2006. Why did modern human populations disperse from Africa ca. 60,000 years ago? A new model. Proc Natl Acad Sci U S A. 103(25):9381–9386. doi:10.1073/pnas.0510792103. 16772383 PMC1480416

[jkaf019-B70] Messer PW , Petrov DA. 2013. Frequent adaptation and the McDonald-Kreitman test. Proc Natl Acad Sci U S A. 110(21):8615–8620. doi:10.1073/pnas.1220835110. 23650353 PMC3666677

[jkaf019-B71] Miles A , Rodrigues MF, Ralph P, Kelleher J, Schelker M, Pisupati R, Rae S, Millar T. 2024. scikit-allel: v1.3.8 (v1.3.8). doi:10.5281/ZENODO.10876220.

[jkaf019-B72] Moorjani P , Amorim CEG, Arndt PF, Przeworski M. 2016. Variation in the molecular clock of primates. Proc Natl Acad Sci U S A. 113(38):10607–10612. doi:10.1073/pnas.1600374113. 27601674 PMC5035889

[jkaf019-B73] Morales-Arce AY , Johri P, Jensen JD. 2022. Inferring the distribution of fitness effects in patient-sampled and experimental virus populations: two case studies. Heredity (Edinb). 128(2):79–87. doi:10.1038/s41437-021-00493-y. 34987185 PMC8728706

[jkaf019-B32] Nicolaisen LE , Desai MM. 2012. Distortions in genealogies due to purifying selection. Mol Biol Evol. 29(11):3589–3600. doi:10.1093/molbev/mss170. 22729750

[jkaf019-B74] Nicolaisen LE , Desai MM. 2013. Distortions in genealogies due to purifying selection and recombination. Genetics. 195(1):221–230. doi:10.1534/genetics.113.152983. 23821597 PMC3761303

[jkaf019-B75] Nielsen R . 2005. Molecular signatures of natural selection. Annu Rev Genet. 39(1):197–218. doi:10.1146/annurev.genet.39.073003.112420. 16285858

[jkaf019-B76] Nielsen R , Williamson S, Kim Y, Hubisz MJ, Clark AG, Bustamante C. 2005. Genomic scans for selective sweeps using SNP data. Genome Res. 15(11):1566–1575. doi:10.1101/gr.4252305. 16251466 PMC1310644

[jkaf019-B77] Pavlidis P , Jensen JD, Stephan W. 2010. Searching for footprints of positive selection in whole-genome SNP data from nonequilibrium populations. Genetics. 185(3):907–922. doi:10.1534/genetics.110.116459. 20407129 PMC2907208

[jkaf019-B78] Poh YP , Domingues V, Hoekstra HE, Jensen JD. 2014. On the prospect of identifying adaptive loci in recently bottlenecked populations. PLoS One. 9(11):e110579. doi:10.1371/journal.pone.0110579. 25383711 PMC4226487

[jkaf019-B79] Pollard KS , Hubisz MJ, Rosenbloom KR, Siepel A. 2010. Detection of nonneutral substitution rates on mammalian phylogenies. Genome Res. 20(1):110–121. doi:10.1101/gr.097857.109. 19858363 PMC2798823

[jkaf019-B80] Przeworski M . 2002. The signature of positive selection at randomly chosen loci. Genetics. 160(3):1179–1189. doi:10.1093/genetics/160.3.1179. 11901132 PMC1462030

[jkaf019-B81] Sackman A , Harris R, Jensen JD. 2019. Inferring demography and selection in organisms characterized by skewed offspring distributions. Genetics. 211(3):1019–1028. doi:10.1534/genetics.118.301684. 30651284 PMC6404255

[jkaf019-B82] Sakharkar MK , Chow VT, Kangueane P. 2004. Distributions of exons and introns in the human genome. In Silico Biol. 4(4):387–393. doi:10.3233/ISB-00142. 15217358

[jkaf019-B83] Sayers EW , Bolton EE, Brister JR, Canese K, Chan J, Comeau DC, Connor R, Funk K, Kelly C, Kim S, et al 2022. Database resources of the National Center for Biotechnology Information. Nucleic Acids Res. 50(D1):D20–D26. doi:10.1093/nar/gkab1112. 34850941 PMC8728269

[jkaf019-B84] Schiffels S , Durbin R. 2014. Inferring human population size and separation history from multiple genome sequences. Nat Genet. 46(8):919–925. doi:10.1038/ng.3015. 24952747 PMC4116295

[jkaf019-B85] Schneider A , Charlesworth B, Eyre-Walker A, Keightley PD. 2011. A method for inferring the rate of occurrence and fitness effects of advantageous mutations. Genetics. 189(4):1427–1437. doi:10.1534/genetics.111.131730. 21954160 PMC3241409

[jkaf019-B86] Schrider DR , Shanku AG, Kern AD. 2016. Effects of linked selective sweeps on demographic inference and model selection. Genetics. 204(3):1207–1223. doi:10.1534/genetics.116.190223. 27605051 PMC5105852

[jkaf019-B87] Siepel A , Bejerano G, Pedersen JS, Hinrichs AS, Hou M, Rosenbloom K, Clawson H, Spieth J, Hillier LW, Richards S, et al 2005. Evolutionarily conserved elements in vertebrate, insect, worm, and yeast genomes. Genome Res. 15(8):1034–1050. doi:10.1101/gr.3715005. 16024819 PMC1182216

[jkaf019-B88] Simkin A , Bailey J, Theurkauf B, Gao FB, Jensen JD. 2014. Inferring the evolutionary history of primate miRNA binding sites: overcoming motif counting biases. Mol Biol Evol. 31(7):1894–1901. doi:10.1093/molbev/msu129. 24723422 PMC4069616

[jkaf019-B89] Soni V , Jensen JD. 2024. Temporal challenges in detecting balancing selection from population genomic data. G3 (Bethesda). 14(6):jkae069. doi:10.1093/g3journal/jkae069. 38551137 PMC11152078

[jkaf019-B90] Soni V , Johri P, Jensen JD. 2023. Evaluating power to detect recurrent selective sweeps under increasingly realistic evolutionary null models. Evolution. 77(10):2113–2127. doi:10.1093/evolut/qpad120. 37395482 PMC10547124

[jkaf019-B91] Soni V , Pfeifer SP, Jensen JD. 2024. The effects of mutation and recombination rate heterogeneity on the inference of demography and the distribution of fitness effects. Genome Biol Evol. 16(2):evae004. doi:10.1093/gbe/evae004. 38207127 PMC10834165

[jkaf019-B92] Soni V , Terbot J, Jensen JD. 2024. Population genetic considerations regarding the interpretation of within-patient SARS-CoV-2 polymorphism data. Nat Commun. 15(1):3240. doi:10.1038/s41467-024-46261-4. 38627371 PMC11021480

[jkaf019-B93] Soni V , Terbot JW, Versoza CJ, Pfeifer SP, Jensen JD. 2024. A whole-genome scan for evidence of recent positive and balancing selection in aye-ayes (*Daubentonia madagascariensis*) utilizing a well-fit evolutionary baseline model. BioRxiv 622667. 10.1101/2024.11.08.622667, preprint: not peer reviewed. PMC12239616 40208178

[jkaf019-B94] Stephan W . 1995. An improved method for estimating the rate of fixation of favorable mutations based on DNA polymorphism data. Mol Biol Evol. 12(5):959–962. doi:10.1093/oxfordjournals.molbev.a040274. 7476143

[jkaf019-B95] Tajima F . 1989. Statistical method for testing the neutral mutation hypothesis by DNA polymorphism. Genetics. 123(3):585–595. doi:10.1093/genetics/123.3.585. 2513255 PMC1203831

[jkaf019-B96] Tataru P , Bataillon T. 2020. polyDFE: inferring the distribution of fitness effects and properties of beneficial mutations from polymorphism data. In: Dutheil JY, editor. Statistical Population Genomics. New York (NY): Springer US. Vol. 2090, p. 125–146. 10.1007/978-1-0716-0199-0_6 31975166

[jkaf019-B97] Tennessen JA , Bigham AW, O’Connor TD, Fu W, Kenny EE, Gravel S, McGee S, Do R, Liu X, Jun G, et al 2012. Evolution and functional impact of rare coding variation from deep sequencing of human exomes. Science. 337(6090):64–69. doi:10.1126/science.1219240. 22604720 PMC3708544

[jkaf019-B98] Terbot J , Cooper B, Good J, Jensen JD. 2023. A simulation framework for modeling the within-patient evolutionary dynamics of SARS-CoV-2. Genome Biol Evol. 15(11):evad204. doi:10.1093/gbe/evad204. 37950882 PMC10664409

[jkaf019-B99] Terbot JW , Johri P, Liphardt SW, Soni V, Pfeifer SP, Cooper BS, Good JM, Jensen JD. 2023. Developing an appropriate evolutionary baseline model for the study of SARS-CoV-2 patient samples. PLoS Pathog. 19(4):e1011265. doi:10.1371/journal.ppat.1011265. 37018331 PMC10075409

[jkaf019-B100] Terbot JW , Soni V, Versoza CJ, Pfeifer SP, Jensen JD. 2025. Inferring the demographic history of aye-ayes (*Daubentonia madagascariensis*) from high-quality, whole-genome, population-level data. Genome Biol Evol. 17(1):evae281. doi:10.1093/gbe/evae281. 39749927 PMC11746965

[jkaf019-B101] Terhorst J , Kamm JA, Song YS. 2017. Robust and scalable inference of population history from hundreds of unphased whole genomes. Nat Genet. 49(2):303–309. doi:10.1038/ng.3748. 28024154 PMC5470542

[jkaf019-B102] The 1000 Genomes Project Consortium; Auton A, Brooks LD, Durbin RM, Garrison EP, Kang HM, Korbel JO, Marchini JL, McCarthy S, McVean GA, et al 2015. A global reference for human genetic variation. Nature. 526(7571):68–74. doi:10.1038/nature15393. 26432245 PMC4750478

[jkaf019-B103] Thornton K . 2003. Libsequence: a C++ class library for evolutionary genetic analysis. Bioinformatics. 19(17):2325–2327. doi:10.1093/bioinformatics/btg316. 14630667

[jkaf019-B104] Wang K , Mathieson I, O’Connell J, Schiffels S. 2020. Tracking human population structure through time from whole genome sequences. PLoS Genet. 16(3):e1008552. doi:10.1371/journal.pgen.1008552. 32150539 PMC7082067

[jkaf019-B105] Wang RJ , Al-Saffar SI, Rogers J, Hahn MW. 2023. Human generation times across the past 250,000 years. Sci Adv. 9(1):eabm7047. doi:10.1126/sciadv.abm7047. 36608127 PMC9821931

[jkaf019-B106] Wiehe TH , Stephan W. 1993. Analysis of a genetic hitchhiking model, and its application to DNA polymorphism data from *Drosophila melanogaster*. Mol Biol Evol. 10(4):842–854. doi:10.1093/oxfordjournals.molbev.a040046. 8355603

[jkaf019-B107] Williamson SH , Hernandez R, Fledel-Alon A, Zhu L, Nielsen R, Bustamante CD. 2005. Simultaneous inference of selection and population growth from patterns of variation in the human genome. Proc Natl Acad Sci U S A. 102(22):7882–7887. doi:10.1073/pnas.0502300102. 15905331 PMC1142382

